# Reduction of meckelin leads to general loss of cilia, ciliary microtubule misalignment and distorted cell surface organization

**DOI:** 10.1186/2046-2530-3-2

**Published:** 2014-01-31

**Authors:** Tyler Picariello, Megan Smith Valentine, Junji Yano, Judith Van Houten

**Affiliations:** 1 Department of Biology, University of Vermont, 120A Marsh Life Science Bldg, Burlington, VT 05405, USA

## Abstract

**Background:**

Meckelin (MKS3), a conserved protein linked to Meckel Syndrome, assists in the migration of centrioles to the cell surface for ciliogenesis. We explored for additional functions of MKS3p using RNA interference (RNAi) and expression of FLAG epitope tagged protein in the ciliated protozoan *Paramecium tetraurelia*. This cell has a highly organized cell surface with thousands of cilia and basal bodies that are grouped into one or two basal body units delineated by ridges. The highly systematized nature of the *P. tetraurelia* cell surface provides a research model of MKS and other ciliopathies where changes in ciliary structure, subcellular organization and overall arrangement of the cell surface can be easily observed. We used cells reduced in *IFT88* for comparison, as the involvement of this gene’s product with cilia maintenance and growth is well understood.

**Results:**

FLAG-MKS3p was found above the plane of the distal basal body in the transition zone. Approximately 95% of those basal bodies observed had staining for FLAG-MKS3. The RNAi phenotype for *MKS3* depleted cells included global shortening and loss of cilia. Basal body structure appeared unaffected. On the dorsal surface, the basal bodies and their associated rootlets appeared rotated out of alignment from the normal anterior-posterior rows. Likewise, cortical units were abnormal in shape and out of alignment from normal rows. A GST pull down using the MKS3 coiled-coil domain suggests previously unidentified interacting partners.

**Conclusions:**

Reduction of MKS3p shows that this protein affects development and maintenance of cilia over the entire cell surface. Reduction of MKS3p is most visible on the dorsal surface. The anterior basal body is attached to and moves along the striated rootlet of the posterior basal body in preparation for duplication. We propose that with reduced MKS3p, this attachment and guidance of the basal body is lost. The basal body veers off course, causing basal body rows to be misaligned and units to be misshapen. Rootlets form normally on these misaligned basal bodies but are rotated out of their correct orientation. Our hypothesis is further supported by the identification of novel interacting partners of MKS3p including a kinetodesmal fiber protein, KdB2.

## Background

Ciliopathies are human disorders caused by abnormalities in the assembly, maintenance or function of cilia and include developmental defects leading to cystic kidneys, vision problems, polydactyly, obesity, encephalocele and even death [1]. In order to improve our understanding of the wide array of cellular processes affected in these disorders, the function and involvement of the genes and gene products involved in ciliopathies should be defined [2-4]. Toward this end, we have investigated the Meckel syndrome type 3 protein (MKS3) in *Paramecium tetraurelia*, a multiciliated cell.

*MKS3* is one of at least three genes commonly associated with the ciliopathy Meckel syndrome (MKS), and has been found to be dysfunctional in other ciliopathy syndromes, including Bardet-Biedl syndrome [5]; cerebellar vermis hypoplasia/aplasia, oligophrenia, ataxia, coloboma and hepatic fibrosis, also known as COACH syndrome [6,7]; and Joubert syndrome [8]. The three most common characteristics of MKS are renal dysplasia, encephalocele and polydactyly [9,10]. The MKS disease is autosomal recessive and has high occurrence rates in Finland, the Middle East, North Africa and Asia [9-12].

Recently, it was shown that MKS3 is a component of a multiprotein complex that contributes to the function of the transition zone to separate the ciliary compartment from the rest of the cell [13]. Other cellular disruptions caused by a reduction of MKS3 have not been closely examined, such as changes in the subsurface scaffolding or in cell surface polarity and surface organization.

*P. tetraurelia* have large numbers of cilia and basal bodies arranged in polarized rows of hexagonal cortical units whose shape is created by a ridge of surface membrane covering an outer lattice. Each cortical unit contains one or two basal bodies. There is one cilium in each of these polarized cortical units (even in units with two basal bodies), as well as one parasomal sac (site of endo- and exocytosis) [14-16], and secretory vesicles (trichocysts) at the apex of the ridges [17-19]. Basal bodies have rootlets emanating from them in a stereotypical orientation relative to the anterior–posterior axis of the cell. The rootlets most likely help secure basal bodies and distribute the forces generated by the beating cilia [20,21]. Units with one basal body have a striated rootlet coursing anteriorly and a transverse and postciliary rootlet projecting in the 5 and 7 o’clock directions, respectively. In units with two basal bodies, the posterior basal body has a cilium and all three rootlets, and the anterior basal body has no cilium and only a transverse microtubule [19,21].

The division process in *Paramecium* is complex and begins with basal body duplication. As the cell elongates, it accommodates for new basal bodies and cilia. The organization of the cortical units and their contents are determined by the “old,” already existing units [22]. Disrupting this organization or development of these cortical units causes growth arrest and even cell death [19,23,24]. It is this highly repetitive patterned surface organization that allows identification of subtle changes in ciliary and surface organization in *P. tetraurelia*.

The results of epitope tagging and RNA interference (RNAi) to perturb the ciliopathy protein MKS3 in *P. tetraurelia* presented herein suggest previously unidentified roles for this protein in maintaining the cell and ciliary membrane surface and cytoskeletal organization. Tagged MKS3 protein is consistently found above the plane of the basal body, in the transition zone of *Paramecium*, which spans from the proximal surface of the epiplasm to the ciliary necklace [25,26]. Dawe and others [27] have published observations of short and missing cilia upon reduction of *MKS3* mRNA levels, which are similar to our findings of short and missing cilia with RNAi. Our study shows new findings, most notably that reduction of *MKS3* causes misalignment of longitudinal rows of basal bodies, rotation of the orientation of basal bodies with their microtubule rootlets and distortion of the cell and ciliary surfaces. We also have evidence of new potential interacting partners of MKS3 relevant to these microtubule rootlets, suggesting important interactions of MKS3 with these structures. The depletion of intraflagellar transport 88 (*IFT88*) mRNA, used as a control to observe global ciliary loss, causes short and missing cilia but does not cause disarray of basal body rows or of the cell surface. We propose that MKS3p in *Paramecium* acts as a transient guide in the movement of basal bodies prior to duplication through an interaction with the microtubule rootlet system and that its localization at the base of the cilium is consistent with an involvement at the transition zone as a filter.

## Methods

### Stocks, cultures and chemicals

Cells (stock 51s *P. tetraurelia*, sensitive to killer) were grown in wheat grass medium inoculated with *Klebsiella pneumoniae* or *Aerobacter aerogenes* (adapted from [28]). All chemicals were obtained from Sigma-Aldrich (St Louis, MO, USA) unless otherwise noted.

### Sequence analysis and construct design

BLAST searches in the *Paramecium* annotated genome were completed using the human sequence for *TMEM67* (Q5HYA8) for *MKS3* and the human *IFT88* (NP_783195) and mouse *Tg737* (NP_033402) sequences for *IFT88* orthologs. Searches identified GSPATG00015939001 as a potential ortholog for *MKS3*, which was used to create the RNAi construct. Five potential orthologs (GSPATG00038505001, GSPATG00021390001, GSPATG00011771001, GSPATG00022644001 and GSPATG00039556001) were identified for *IFT88*. The construct to target *IFT88* mRNA was designed from GSPATG00038505001. Homology of these genes to those in other organisms is shown in Additional file 1: Tables S1 and S2.

All constructs were created from genomic DNA, which was collected by organic extraction. Briefly, 100 μl of cells were mixed 1:1 with denaturing buffer (Promega, Madison, WI, USA), mixed 1:1 with phenol:chloroform:isoamyl alcohol (25:24:1) and centrifuged for 5 minutes at 12,000 × *g* (Centrifuge 5424; Eppendorf, Hauppauge, NY, USA). The aqueous phase was removed, mixed 1:1 with chloroform:isoamyl alcohol (24:1) and spun again. The DNA was precipitated 2:1 with cold isopropanol for 20 minutes at -20°C and spun for 10 minutes at 4°C (Centrifuge 5424). Pellets were rinsed twice with 75% ethanol, dried and resuspended in water.

### FLAG-tag of *MKS3*

To localize MKS3p, we added the coding sequence for a threefold repeated FLAG sequence (DYKDDDDK) to the 5′ end of the genomic DNA sequence for GSPATG00015939001 in the pPXV plasmid using the restriction enzymes ApaI and SacI (USB/Affymetrix, Cleveland, OH, USA). These cut sites were created using large primers to add them to either end of the sequence: forward (5′-gcggggcccatgctaatttatatcg-3′) and reverse (5′-cgcgagctctcatattagaaaccttttgtc-3′). Platinum*Pfx* Polymerase (Invitrogen/Life Technologies, Grand Island, NY, USA) was used per the vendor’s instructions to amplify the sequence. A total of 75 ng of genomic DNA was used in each PCR: 94°C for 5 minutes; five cycles of 94°C for 1 minute, 40°C for 1 minute and 68°C for 3 minutes; five cycles of 94°C for 1 minute, 48°C for 1 minute and 68°C for 3 minutes; ten cycles of 94°C for 1 minute, 58°C for 1 minute and 68°C for 3 minutes; seventeen cycles of 94°C for 1 minute, 65°C for 1 minute and 68°C for 3 minutes; and one cycle of 68°C for 15 minutes (Techne TC-4000 Thermal Cycler; Krackeler Scientific, Albany, NY, USA). The products were cleaned using the PrepEasy Gel Extraction Kit (Affymetrix). The resulting DNA was treated with restriction enzymes, cleaned again using the PrepEasy Gel Extraction Kit and ligated into the pPXV-5′-3xFLAG plasmid using the Ligate-IT Kit (Affymetrix). The mixture was then transformed into OneShot competent cells (Invitrogen/Life Technologies), and the resulting colonies were screened for positives. Positive clones were sequenced at the Vermont Cancer Center DNA Analysis Facility (University of Vermont, Burlington, VT, USA).

### Plasmid injection

Approximately 200 μg of pPXV-3xFLAG-*MKS3* was linearized with NotI (Affymetrix) overnight at 37°C and then cleansed using an organic extraction method modified from that described earlier. This procedure required two washes in phenol:chloroform:isoamyl alcohol (25:24:1) followed by two washes of chloroform:isoamyl alcohol (24:1). The final pellet was resuspended in 50 μl of MilliQ water (EMD Millipore, Billerica, MA, USA), and the concentration was checked using a spectrophotometer (Agilent Technologies, Santa Clara, CA, USA). The sample was spun at 16,000 × *g* (Eppendorf Centrifuge 5424) for 10 minutes to pellet debris. The top 45 μl was carefully removed and placed in a fresh RNase/DNase-free 1.5-ml Eppendorf tube and again dried in a speed vac. The final pellet was resuspended in MilliQ water to obtain a concentration between 3 and 9 μg/μl and stored at 4°C until injection.

Approximately 20 cells which had recently undergone autogamy were placed under high-temperature silicon oil to immobilize them. Approximately 5 to 50 pg of the plasmid was injected into the macronucleus of each cell using a pulled capillary and a Narishige micromanipulator (Narishige International USA, East Meadow, NY, USA). Individual injected cells were transferred to 750 μl of inoculated culture fluid in depression slides and incubated in a humidifying chamber at RT for 2 days, allowing the cells to recover and divide. Cells were then transferred to test tubes with inoculated culture fluid and maintained at 15°C as individual clones. Genomic DNA was extracted from the clone cultures as described previously (see Sequence analysis and construct design text section) and tested by PCR using plasmid-specific primers: the forward primer for the plasmid pPXV (5′-taagatgaatggaatataatg-3′) and a reverse primer (5′-gaaaacccaagccaatcaatac-3′), which was sequence-specific for *MKS3*. DNA (1 μl, approximately 400 ng) was used in each PCR: one cycle at 95°C for 5 minutes followed by 30 cycles at 95°C for 1 minute, 40°C for 1 minute and 72°C for 3 minutes followed by one 15-minute cycle at 72°C.

### Localization, visualization and analysis of FLAG-MKS3p

We tested small cultures of individual clones to ascertain whether the cells expressed the protein and where it was localized. A 10-ml culture of injected cells was added to 50 ml of inoculated culture fluid and grown at 22°C for approximately 48 to 72 hours. The cells were immunostained and imaged as described below. Images were analyzed for colocalization using softWoRx Pro software (Applied Precision, Issaquah, WA, USA). Experiments were repeated five times.

To isolate pellicle membrane and whole cilia membrane, wild-type (stock 51s *P. tetraurelia*) cells expressing FLAG (control) or FLAG-MKS3 (Test) were maintained in large cultures (3 to 6 L of culture fluid) at 22°C until a density of 8,000 to 12,000 cells/ml was achieved. For pellicular membrane, cells were harvested as described previously [29]. In separate experiments, cilia were separated from cell bodies and collected as previously described [30] up to the point of separation of the ciliary membrane from the axoneme. Protein concentrations were determined using a bicinchoninic acid protein assay (Thermo Scientific, Pittsburgh, PA, USA) and equalized between the test and control. Samples were separated on a 12% SDS-PAGE gel after adding 1 μl of β-mercaptoethanol and boiling for 5 minutes. One hundred micrograms of pure pellicular membrane and 400 μg of whole cilia were loaded, along with 10 μl of a Pierce Biotechnology three-color prestained protein molecular weight marker (Thermo Scientific). Proteins were transferred onto nitrocellulose membrane (Pall Gelman Versapor; Krackeler Scientific, Albany, NY, USA) and blocked for 1 hour using 5% nonfat dry milk, 2% Telost gelatin from fish, 3% normal goat serum (Vector Laboratories, Burlingame, CA, USA), in Tris-buffered saline Tween 20 (TBS-T) (15 mM Tris-Cl, 140 mM NaCl, 0.1% v/v Tween 20, pH 7.5). Blots were probed with a 1:2,500 dilution of rabbit Anti-FLAG M2 clone or 1:10,000 mouse anti-tubulin in the blocking buffer. Blots were incubated overnight while rocking at 4°C. Buffers were removed, the blots were rinsed three times in TBS-T and then incubated for 1 h in 1:10,000 goat anti-rabbit or anti-mouse alkaline phosphatase (AP)-conjugated secondary antibody. Blots were rinsed again four times in TBS-T for 15 minutes for each wash and developed using nitroblue tetrazolium/5-bromo-4-chloro-3′-indolyl phosphate AP (Moss, Inc, Pasadena, MD, USA).

### RNAi by feeding construct

Constructs for RNAi were created from genomic DNA using the following primers: *MKS3* forward, 5′-gaaaacccaagccaatcaatac-3′ and reverse, 5′-ggtcgacaatctgaaggataag-3′; and *IFT88* forward, 5′-caattaaggaaaaccacctg-3′ and reverse, 5′-aaaactaacaggattgtcatct-3′. All PCR conditions began with an initial step at 95°C for 5 minutes and ended with a final stage at 72°C for 20 minutes. The *MKS3* RNAi construct was amplified by 30 cycles at 95°C for 1 minute, 52°C for 1 minute and 72°C for 2 minutes. The *IFT88* construct was amplified by five cycles at 95°C for 1 minute, 47°C for 1 minute and 72°C for 2.25 minutes; followed by twenty-five cycles at 95°C for 1 minute, 50°C for 1 minute and 72°C for 2.25 minutes (Techne Thermal Cycler; Bibby Scientific, Burlington, NJ, USA). The final PCR products were analyzed on 0.75% or 1.0% agarose gel (Invitrogen/Life Technologies) and visualized with ethidium bromide. Resulting PCR products were cloned directly into pCR2.1-TOPO vector (Invitrogen/Life Technologies), transformed into OneShot cells (Invitrogen/Life Technologies), and sequenced. Correct sequences were cut from the pCR2.1-TOPO vector and ligated into the double-T7 promoter vector L4440 (AddGene, Cambridge, MA, USA) using the Ligate-IT Kit (USB/Affymetrix) as per the kit instructions. *Escherichia coli* strain Ht115 (DE3), which lacks RNaseIII, was transformed with 50 ng of plasmid DNA for either *MKS3* or *IFT88*. As a control, Ht115 cells were transformed with L4440 with no insert. Bacterial cultures were maintained with tetracycline (12.5 μg/ml) and ampicillin (AMP) (100 μg/ml).

### RNAi by feeding

Overnight cultures of Ht115(DE3) transformed with RNAi or control plasmids were used to inoculate 50 ml of LB-AMP (100 μg/mL) and grown until the 595-nm optical density reached 0.3 to 0.4, at which point isopropyl β-D-1-thiogalactopyranoside (IPTG) (RPI, Mount Prospect, IL, USA) was added to a final concentration of 0.125 mg/ml. Cultures were incubated with shaking for 3 hours at 37°C to induce the production of double-stranded RNA. Paramecia that had recently undergone autogamy were collected by centrifugation and resuspended in 10 ml of Dryl’s solution (1 mM Na_2_HPO_4_, 1 mM NaH_2_PO_4_, 1.5 mM CaCl_2_, 2 mM Na-citrate, pH 6.8) to purge bacteria from their surfaces and food vacuoles.

The induced bacteria were collected by centrifugation at 4,000 × *g* (Beckman J2-21 centrifuge, JA-14 rotor; Beckman Coulter, Brea, CA, USA) at 4°C and resuspended in 100 mL of wheat culture medium containing an additional 8 μg/mL stigmasterol, 0.125 mg/mL IPTG (RPI), and 100 μg/ml AMP. Approximately 50 to 100 of the purged paramecia were added to the control culture. In the case of the *MKS3* and *IFT88* RNAi cultures, 4,000 and 8,000 cells were added to 100 ml, respectively. Cultures were maintained at 28°C, and after 24 hours, an additional 0.125 mg/ml of IPTG (RPI) and 800 μg of stigmasterol were added. Growth rates of cultures were determined by counting cells at 24, 48 and 72 hours of growth. All experiments were repeated a minimum of three time and all cultures were harvested or observed after approximately 72 hours of growth unless noted otherwise.

### Immunofluorescence

Cultured cells (100 ml) were collected by centrifugation (Damon IEC Division Clinical Centrifuge, Needham Heights, MA, USA) and rinsed twice in 100 ml of Dryl’s solution. The cell volume was reduced to approximately 100 μl in a 1.5-ml Eppendorf tube before 1 ml of PHEM and 0.1% or 0.5% Triton X-100) was added. Cells were undisturbed for 1 to 4 minutes, then spun at 250 × *g* (Damon IEC Division Clinical Centrifuge) and then the supernatant was removed and the pellet (cells) was mixed with 1 ml of fixation buffer (2% or 4% paraformaldehyde (Electron Microscopy Sciences, Hatfield, PA, USA), 2 mM NaH_2_PO_4_•H_2_O, 8 mM Na_2_HPO_4_, 150 mM NaCl, pH 7.5). Samples were undisturbed for 10 minutes or rocked for 1 hour at room temperature (RT) and washed three times in 1 ml of blocking buffer (2 mM NaH_2_PO_4_•H_2_O, 8 mM Na_2_HPO_4_, 150 mM NaCl, 10 mM EGTA, 2 mM MgCl_2_, 0.1% Tween 20, 1% or 3% bovine serum albumin (BSA), pH 7.5).

Primary antibodies for the immunostaining for localization were as follows: FLAG-MKS3: mouse anti-FLAG, M2 clone at a 1:300 dilution (Sigma-Aldrich) and anti-centrin at a 1:1,000 dilution (anti-*Tetrahymena* centrin, gift from Mark Winey, University of Colorado, Boulder, CO, USA). For ciliary measurements, we used mouse anti-α-tubulin at a dilution of 1:200 (Sigma-Aldrich). For visualization of basal bodies, we used anti-centrin at a dilution of 1:1,000. For cortical unit visualization, we used anti-2F12 at a dilution of 1:200 (gift from Jean Cohen, Gif-sur-Yvette, France). For the visualization of the kinetodesmal fibers (KDFs), we used anti-KDF at a 1:400 dilution (gift from Janine Beisson, Centre de Génétique Moléculaire, Gif-sur-Yvette, France) and anti-Glu-α-tubulin at a 1:500 dilution (Synaptic Systems, Göttingen, Germany). Primary antibodies in 100 μl of blocking buffer were mixed with the cells and rocked at RT for 1 hour. Cells were washed three times in blocking buffer or wash buffer (2 mM NaH_2_PO_4_•H_2_O, 8 mM Na_2_HPO_4_, 150 mM NaCl, 0.1% Tween 20, 1% BSA, pH 7.5). The cells were mixed with 100 μl of blocking buffer with a 1:200 dilution of secondary antibodies. Secondary antibodies (Molecular Probes/Invitrogen, Grand Island, NY, USA) included Alexa Fluor 488 or 555 goat anti-mouse and Alexa Fluor 488 or 568 goat anti-rabbit. After 30 minutes to 1 hour of incubation while rocking, cells were washed three to five times with blocking or wash buffer and, to the final 20 μl of cells, one drop (approximately 15 μl) of VECTASHIELD mounting medium (Vector Laboratories, Burlingame, CA, USA) was added. Tubes were wrapped in aluminum foil and stored at 4°C until use.

Imaging of the immunostained cells was done using a DeltaVision Restoration Microscopy System (Applied Precision), consisting of an inverted Olympus IX70 microscope (Olympus America, Center Valley, PA, USA) and a Kodak CH350E camera (Rochester, NY, USA). Prepared cells (7 μl) were placed under a glass coverslip and imaged at 20°C to 22°C using either a PlanApo 60× or 100×/1.40 oil-immersion lens objective and deconvolved and analyzed using softWoRx Pro software.

Colocalization of FLAG-MKS3 and centrin (basal bodies) was analyzed using softWoRx Pro software or ImageJ software [31]. Eleven cells were analyzed for the colocalization of these two proteins. To examine the staining patterns and calculate the number of basal bodies with FLAG-MKS3 staining, 15 μm × 15 μm grids were chosen from both the ventral and dorsal surfaces of each of three cells. Basal bodies within that grid were counted, and we noted whether they had FLAG-MKS3 staining. A total of 463 basal bodies were analyzed on these three cells.

### Scanning electron microscopy

RNAi cultured cells (200 ml) were collected by brief centrifugation at 800 × *g* (Damon IEC Division Clinical Centrifuge), washed twice in Dryl’s solution and fixed as described by Lieberman *et al*. [32]. After critical point drying, coverslips were glued onto an aluminum chuck using colloidal graphite cement and allowed to dry in a desiccator overnight. The samples were sputter-coated and stored in a desiccator until imaged using a JSM-6060 scanning electron microscope (JEOL USA, Peabody, MA, USA).

### Transmission electron microscopy

RNAi cultured cells (100 ml) were collected by brief centrifugation at 800 × *g* (Damon IEC Division Clinical Centrifuge) and washed twice in 100 ml of Dryl’s solution, then approximately 100 μL of the cell pellet was removed and placed in 1.5-ml Eppendorf tubes. One milliliter of Fixation Solution A (1% gluteraldehyde (Electron Microscopy Sciences), 0.05 M sodium cacodylate, pH 7.2) was added, rocked for 30 minutes on ice and washed three times for 10 minutes under the same conditions. Cells were resuspended in postfix Solution B (1% gluteraldehyde (Electron Microscopy Sciences), 0.05 M sodium cacodylate buffer, 1% osmium tetroxide, pH 7.2) and again washed and rinsed as described above. Cells were preembedded in 2% agarose gel (Invitrogen/Life Technologies) in 0.05 M sodium cacodylate buffer and allowed to set, then sliced into 1 mm × 1 mm blocks. Blocks were placed in glass vials with 50% ethanol and rocked on a specimen rotator for 30 minutes during each of the following washes: ethanol at concentrations of 50%, 70% and 90% and two times at 100%, with both of the latter in propylene oxide. Cells were left overnight on a specimen rotator in 1:1 propylene oxide and Spurr’s solution (Electron Microscopy Sciences). The next day, samples were placed in fresh Spurr’s solution for 6 hours and placed in flat embedding molds with fresh Spurr’s solution at 60°C for 48 hours. Sections were cut to 90-nm thickness, placed on copper 200-mesh grids and contrasted on droplets of 2% uranyl acetate in 50% ethanol for 6 minutes followed by lead citrate (120 mM sodium citrate, 2.66% lead nitrate and 0.65% sodium hydroxide in water) for 4 minutes. Sections were imaged using a JEM-1210 electron microscope (JEOL USA). These studies were repeated three times.

### Glutathione *S*-transferase pull-down and mass spectrometry analysis

The coiled-coil domain of MKS3 was expressed with a glutathione *S*-transferase (GST) tag for use in a GST pull-down assay. The construct was created by amplifying positions +2,183 to +2,273 of GSPATG00015939001 using the following forward and reverse primers, respectively: 5′-gcgggatccatgaattttgtcgatctc-3′ and 5′-gcggaattctgatggattttctccatg-3′. The PCR product was treated with BamHI and EcoRI restriction enzymes (New England Biolabs, Ipswich, MA, USA) and cleaned using gel purification and the PrepEase Gel Extraction Kit (Affymetrix, Santa Clara, CA, USA), then ligated into a pGEX-2TK plasmid vector (GE Healthcare Life Sciences, Pittsburgh, PA, USA) using the Ligate-IT Rapid Ligation Kit (Affymetrix). The pGEX-2TK plasmid vector had already been opened using the same restriction enzymes, treated with 1 U of calf intestinal alkaline phosphatase at 37°C for 5 minutes to remove the phosphate groups, followed by heat inactivation with 5 mM Na_2_-ethylenediaminetetraacetic acid at 72°C for 20 minutes. The GST-MKS3 coiled-coil domain and GST were expressed in BL-21 cells and bound to glutathione sepharose beads (GE Healthcare Life Sciences) as described previously [33]. After beads were collected from bacterial cell lysates, they were washed in a 1 M MgCl_2_ buffer to remove bacterial proteins from the GST and GST-MKS3 proteins. Protein-bound beads were stored at 4°C in phosphate-buffered saline for up to 2 weeks.

Stock 51s *P. tetraurelia* cells were cultured and harvested as described previously [34] for whole-cell extract (WCE). Glutathione sepharose beads (GE Healthcare Life Sciences) were prepared by washing three times in LAP200 buffer (50 mM HEPES, 200 mM KCl, 1 mM EGTA, 1 mM MgCl_2_, pH 7.4) buffer with 1% Triton X-100. Washed beads (200 μl) were added to 20 ml of WCE. This precleared WCE was then split in half and incubated with 200 μl of glutathione sepharose beads attached to either GST or GST-MKS3. Beads in the supernatant were allowed to rock on ice at 4°C for 1 hour. Control and test beads were recovered and washed three times in LAP200 buffer with 1% Triton X-100. Samples were run on a 7% to 14% gradient acrylamide gel and silver-stained, then gel slices were trypsin-digested as described previously [34].

Samples were analyzed by liquid chromatography-tandem mass spectrometry (LC-MS/MS) on a linear ion trap LTQ XL Linear Ion Trap Mass Spectrometer (Thermo Fisher Scientific, Asheville, NC, USA). Half the material was loaded onto a 100-μm × 120 mm capillary column packed with MAGIC C18 (5-μm particle size, 20-nm pore size; Michrom Bioresources, Auburn, CA, USA) at a flow rate of 500 nl/min. Peptides were separated by a gradient of 5% to 35% CH_3_CN/0.1% formic acid for 30 minutes, 40% to 100% CH_3_CN/0.1% formic acid for 1 minute and 100% CH_3_CN for 10 minutes.

Product ion spectra were searched using the SEQUEST search engine on Proteome Discoverer 1.4 (Thermo Fisher Scientific) against a curated *P. tetraurelia* database with sequences in forward and reverse orientations. The 13 raw files from control samples and the 13 raw files from test samples were searched as one contiguous input file, and a single result file was generated for each. The database was indexed to allow for full trypsin enzymatic activity, two missed cleavages and peptides between the molecular weights of 350 to 5,000 Da. Search parameters set the mass tolerance at 2 Da for precursor ions and 0.8 Da for fragment ions. The result files were then searched against Scaffold version 4.0.5 software (Proteome Software, Portland, OR, USA). Cross-correlation (xcorr) significance filters were applied to limit the false-positive rates to less than 1% in both data sets. The xcorr values were as follows: (+1): 1.8, (+2): 2.7, (+3): 3.3 and (+4): 3.5. Other filters applied were a minimum peptide cutoff of 2 as well as DeltaCN >0.1. Ultimately, the confidence parameters resulted in 0% false discovery rate at the protein and peptide level for both the control and test results.

## Results

### Sequence analysis

The sequence for *MKS3* in *Paramecium* (GSPATG00015939001, 4e^-57^, 23% identity) was found using the human sequence for *TMEM67* (Q5HYA8) and the annotated *Paramecium* genome (ParameciumDB, http://paramecium.cgm.cnrs-gif.fr/). *Paramecium MKS3* (*TMEM67*) codes for 2,906 nucleic acids and 951 amino acids. The RNAi construct design for *Paramecium MKS3* comprises bases from positions +1,101 to +2,019.

Five potential homologues for *IFT88* were found using the human *IFT88* (NP_783195) and mouse *Tg737* (NP_033402) sequences. The best match was GSPATP00038505001 (e^-146^, 38% identity), which codes for 2,341 nucleic acids and 743 amino acids. The RNAi construct for *Paramecium IFT88* spans positions +48 to +2,121. Using a feature of ParameciumDB to identify potential off-target effects [35], we found that the *MKS3* RNAi plasmid will target only GSPATG00015939001, whereas the RNAi plasmid for *IFT88* will target all five homologues but no other gene sequences outside this gene family. Included in the Supplemental Material are tables comparing *Paramecium IFT88* and *MKS3* (Additional file 1: Table S1 and Table S2) with sequences from other organisms. To further document the conservation of these proteins in *Paramecium*, amino acid alignments of full length and conserved regions in each protein are included in Additional file 2: Figure S1 and Additional file 3: Figure S2.

### FLAG-MKS3p immunostaining and localization

**Figure 1:**
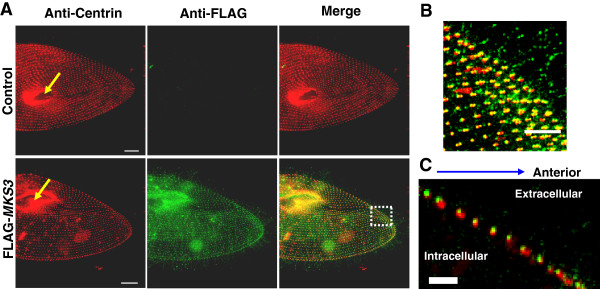
**Localization of FLAG-MKS3p. (A)** Cells expressing FLAG (control) or FLAG-*MKS3* were immunostained with anti-centrin (red) and anti-FLAG (green). Representative cells are shown. The oral grove (mouth) is indicated by yellow arrows. **(B)** FLAG staining in the test cell expressing FLAG-*MKS3* in **(A)** shows a punctate pattern at each basal body with partial colocalization. **(C)** This staining is above the plane of the basal body (see also Additional file 4: Figure S4 for Western blots and Additional file 5: Movie S1 for individual Z sections). Images are stacks of Z sections, with thicknesses of 10 to 15 μm **(A)** and **(B)** or 4 μm **(C)**. Scale bars are 15 μm **(A)**, 5 μm **(B)** and 2 μm **(C)**.

We used a 5′-3xFLAG-tagged (FLAG*-MKS3*) expression vector to produce FLAG-MKS3p to localize the MKS3 protein. Control paramecia were derived from cells that were injected with the empty FLAG vector to confirm that cells were unaffected by the expressed FLAG peptide. Cells were permeabilized, stained with anti-centrin and anti-FLAG and imaged. In Figure 1, images are stacks to ensure that basal bodies and FLAG-MKS3p staining are visible. Cells expressing FLAG or FLAG*-MKS3* showed similar centrin staining patterns across the cell surface (Figure 1A). The control cells show almost no staining by anti-FLAG, but the FLAG*-MKS3*-expressing cells show very clear FLAG staining near the basal bodies and faint staining in the cilia (Figure 1A and arrows in Figure 1C; see also Western blots of the tagged protein from cell membrane and cilia in Additional file 4: Figure S4). Additional file 5: Movie S1 demonstrates this pattern of FLAG-MKS3p staining above the staining of centrin. When scanning through the same cell, starting from the surface, the green FLAG staining can be seen prior to the red staining of the centrin. Figure 1B also demonstrates the FLAG-MKS3p staining at the distal side of the centrin staining, that is, above the staining of the basal body. The anti-centrin antibody recognizes *Tetrahymena* centrin 1, which is homologous to *Paramecium* centrin 2, which is found in the basal body along the shaft [24]. The transition zone of the *Paramecium* cilium has been defined as stretching from the basal body, near the proximal surface of the epiplasm, to the base of the cilium, where the triplets of microtubules become doublets and the central pair of microtubule doublets begins [25,26]. The localization of the MKS3 protein is therefore consistent with that of the transition zone for these cells.

To quantify the basal body and FLAG-MKS3p staining, 15 μm × 15 μm squares on the dorsal and ventral surfaces of three different cells expressing FLAG-*MKS3* were randomly chosen. Of the 463 basal bodies observed, 95.2% ± 2.2% of them had FLAG-*MKS3* staining. These data suggest that where a basal body is present, we expect to find MKS3 protein. To quantify the extent of colocalization of the centrin and FLAG-MKS3p staining, the images were analyzed using softWoRx software to obtain a Pearson’s coefficient (*r*). Eleven FLAG-MKS3p cells showed an average colocalization score of 0.46 ± 0.11 (*r* ± SD), indicating partial colocalization. FLAG-MKS3p staining is clearly seen in the oral groove (Figure 1A, yellow arrows), but we were unable to differentiate individual basal bodies in this region because of their close packing and the spatial limitations of fluorescence microscopy. Therefore, these oral groove basal bodies were not included in our analysis.

### Ultrastructure

We utilize RNAi by feeding of paramecia because of its ease of use and because the creation of knockouts by homologous recombination is not possible. RNAi allows us to observe the cells in a depleted state of a targeted protein quickly and effectively in a wild-type background. RNAi is estimated to be 80% effective [36]. It allowed us to leave variable amounts of the targeted protein in the cells and thereby protect them from lethal effects of complete loss of MKS3. We found that very aggressive RNAi treatment quickly led to cell death. In this way, RNAi had an advantage over gene knockout.

**Figure 2:**
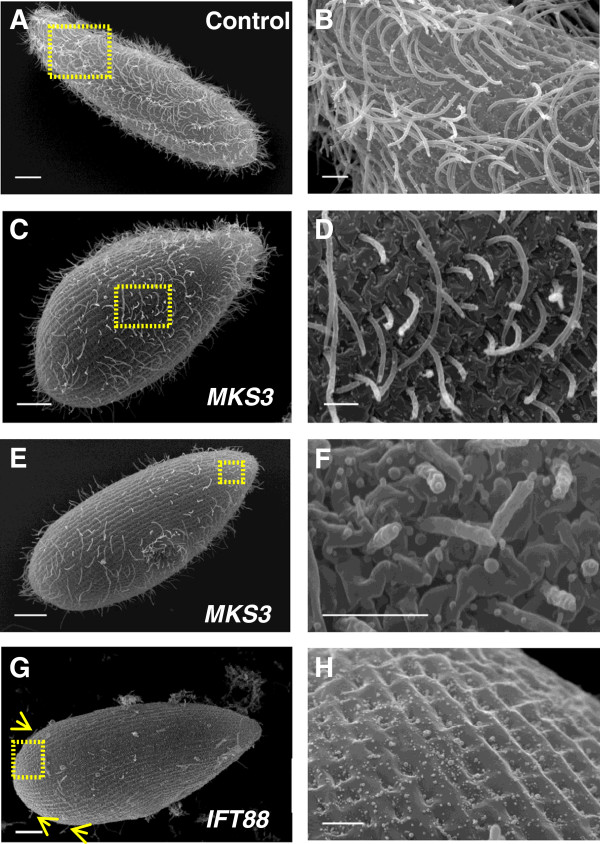
***MKS3-*depleted cells have sparse cilia and distorted cell and ciliary surfaces.** Scanning electron micrographs of representative images of control **(A)** and **(B)**, *MKS3*-depleted **(C)** through **(F)** and *IFT88*-depleted **(G)** and **(H)** cell populations. The cell in **(G)** has been rotated. Whole-cell images of control **(A)**, *MKS3*-depleted **(C)** and **(E)** and *IFT88*-depleted **(G)** cells. Scale bars: 10 μm. The control cell **(A)** is covered in cilia, whereas the *MKS3*-depleted cells show sparse and short cilia **(C)** and **(E)**. The *IFT88*-depleted cell **(G)** has almost no cilia remaining, although a few short cilia are visible (yellow arrows). Higher magnifications of the cell surfaces from **(A)**, **(C)**, **(E)** and **(G)** (yellow boxes) are shown in **(B)**, **(D)**, **(F)** and **(H)**, respectively (scale bars: 2 μm). Control cell surface **(B)** shows cilia arising from the cortical units, whereas the *MKS3*-depleted cells show short and missing cilia **(D)** and **(F)**. Over 50% of the *MKS3*-depleted cells have cilia that are severely misshapen and “blebby.” The cell surfaces of these cells also became heavily wrinkled and distorted **(F)**. The *IFT88*-depleted cell **(H)** shows normal organization of cortical units.

Scanning electron micrographs (SEMs) show that the control cells were covered in cilia and display a highly organized cell surface with one cilium protruding from each cortical unit (Figures 2A and 2B). The cilia on the control cells appear normal, as shown in the representative cell in Figure 2A. The *MKS3*-depleted cells displayed very short and sparse cilia (Figures 2C to 2F) and look dramatically different from the controls. The cilia that are present do not resemble the control cilia. They have wrinkled surfaces and bulges at the tips (Figures 2E and 2F). Of the 23 *MKS3*-depleted cells observed, 56.5% displayed the “blebby” cilia. This was not observed on any of the control cells. The control cell in Figure 2A is fixed with the metachronal wave intact, but the *MKS3*-depleted cell shown in Figure 2B is not, which is far more common. This difference between the two cells is not a consequence of the reduction of *MKS3* mRNA. The disturbance to the cell and cilia surface by reduction of *MKS3* mRNA is more evident at higher magnification (Figures 2D and 2F). We used RNAi to reduce mRNA for *IFT88* that is known to cause loss of cilia by failure of intraflagellar transport (IFT), a mechanism that is specific to ciliary development and maintenance. The *IFT88*-depleted cells display a normally patterned cell surface (Figures 2G and 2H), with very few and short cilia present on the surface of the majority of the cells (Figure 2G, yellow arrows).

*MKS3* RNAi resulted in cells that appear, by SEM examination (Figure 2), to have missing or shortened cilia everywhere on the cell, except in the oral groove. For more details regarding short cilia, refer to Additional file 6. The reduction of *MKS3* using RNAi also caused severe distortions in the cell surface, in contrast to the surfaces of cells depleted of *IFT88*, which show missing cilia but no other disruptions.

Transmission electron microscopy (TEM) was employed to examine the ultrastructure of basal bodies to determine whether they were structurally equivalent in control and *MKS3*-depleted cells. The loss of cilia that we observed was not due to their inability to properly form basal bodies. Cross-sections of basal bodies observed using TEM were measured for both height and width using ImageJ software [31]. No differences were observed between control basal bodies, which were 379.6 ± 42.4 nm in length and 202.9 ± 22.8 nm in width (*n* = 13), and *MKS3*-depleted basal bodies, which were 367.7 ± 35.5 nm long and 191.8 ± 21.9 nm wide (*n* = 14). In addition, no obvious differences in basal body docking were observed.

### Immunofluorescence: basal bodies and cortical units

**Figure 3:**
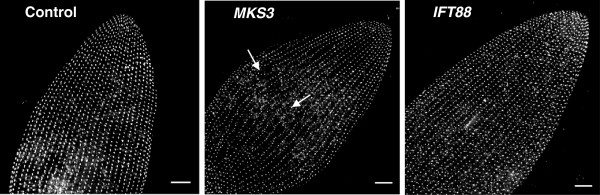
**Basal bodies of *MKS3***-**depleted cells are misaligned primarily at the dorsal midline.** Cells were stained using anti-centrin to visualize the basal bodies. Images represent stacks of Z sections approximately 10 μm thick. The images shown are the dorsal surface of the anterior side of the cell. Basal bodies should be arranged in organized rows, as shown in the control and *IFT88*-depleted cells. The *MKS3*-depleted cell (center panel) shows the basal bodies not aligned and no longer in straight rows (white arrows) at the midline of the cell. Scale bars: 10 μm.

Immunostaining of the *MKS3*-depleted cells with anti-centrin revealed a basal body pattern that differed from that of the control and *IFT88*-depleted cells. The images shown in Figure 3 are stacks of Z-sections approximately 10 μm thick, which we used to ensure all basal bodies could be visualized. The representative views shown are of the anterior dorsal surfaces of the cells. The control cell shows rows of basal bodies that run from anterior to posterior (Figure 3). The basal body rows at the midline of the typical *MKS3*-depleted cell show disorganization and twisting (Figure 3, white arrows). Distortions of rows can be seen elsewhere on the dorsal sides of the cells, but are most commonly observed at the dorsal midline. The control and *IFT88*-depleted cells maintain straight, organized rows.

**Figure 4:**
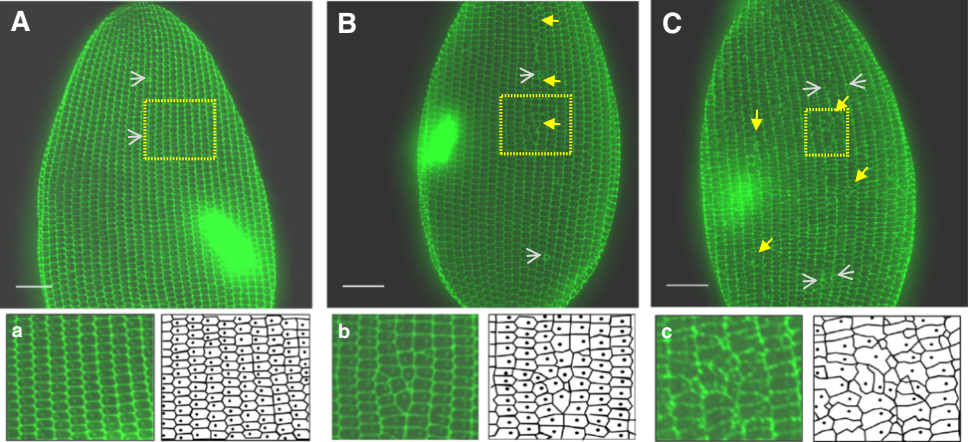
**Disruptions of the cortical units correspond to the areas of basal body misalignment.** Control **(A)** and *MKS3*-depleted cells **(B)** and **(C)** were stained using anti-2F12 to visualize the cortical ridges. These images show the dorsal surface and are stacks of Z sections approximately 10 μm thick. The bright green immunofluorescence spots indicate the oral grooves. The control cell in **(A)** shows the high degree of organization of the outer lattice across the cell surface. The presence of a basal body in each unit can be seen in the enlarged area (**A**(**a**), yellow box) and the traced image below (black dot inside the hexagonal unit). The *MKS3*-depleted cells illustrate insertion of several cortical units forming an abbreviated kinety (**B**(**b**), yellow arrows) or a disruption type caused by the insertion of multiple cortical units, resulting in the formation of a cluster (**C**(**c**), yellow arrows). The contractile vacuole pores are indicated by gray arrows. Scale bars: 10 μm.

In Figure 4, the ridges of the cortical units are highlighted using anti-2F12 at 60× magnification. Light staining of a basal body can be seen at the center of each unit. The dorsal surface of a control cell (Figure 4A) demonstrates the high level of organization of the cortical units. An area of the control cell has been enlarged (yellow box) to better highlight this surface (Figure 4a). The lower images in Figures 4a to 4c have been traced for clarity and are to the right of each image. The contractile vacuole pores are indicated by gray arrows. The two *MKS3*-depleted cells (Figures 4B and 4C) show two major types of differences from the control: an insertion of cortical units that incorporates a short row into another row of units (a kinety) (Figure 4B, yellow arrows) and clustering of basal bodies that should be organized in a row (Figure 4C, yellow arrows). The *MKS3*-depleted cell with the insertion of an abbreviated kinety has a basal body for almost every cortical unit (Figure 4B), whereas the complete surface disruption, the clustering, shows chaotic organization of the cortical units, some of which are missing a basal body (Figure 4C). Of the *MKS3*-depleted cells, 70% show kinety disruptions. Of those 70%, 90% had clusters of basal bodies, as shown in Figure 4C, and 10% had an insertion of a partial kinety row, as shown in Figure 4B. These changes in ridge patterns were always observed on the dorsal surfaces of the cells, often near the midline and never at the extreme poles of the cell, in over 30 control and 70 *MKS3*-depleted cells.

Each cortical unit has one or two basal bodies with corresponding microtubule rootlets. The transverse microtubule (TM) and the postciliary microtubule (PCMs) are oriented in 5 o’clock and 7 o’clock directions, with the anterior end of the cell pointing to 12 o’clock. We examined the orientation of TMs and PCMs using an anti-α-tubulin antibody and the basal bodies using the *Tetrahymena* anti-centrin antibody. The *MKS3-*depleted cells lost most of their cilia, which facilitated the imaging of the basal bodies and the cortical microtubule cytoskeleton. However, the cilia on control cells obscured the image of the cortical microtubules that were visualized with the anti-α-tubulin antibody. Therefore, we used *IFT88*-depleted cells as a control because they lose their cilia but do not lose alignment of basal bodies in orderly rows of cortical units (Figure 3).

**Figure 5:**
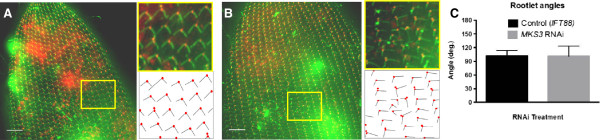
**Microtubule ribbons have an altered orientation in *MKS3 *depleted cells. ***IFT88*-depleted cells **(A)** and *MKS3*-depleted cells **(B)** cells were stained using anti-centrin basal bodies (red) and anti-tubulin microtubule rootlets (green). Images are stacks of Z sections approximately 10 μm thick. All images show the anterior dorsal surface. Areas in **(A)** and **(B)** (yellow box) have been enlarged, and below each is a tracing to depict the basal body (red) and the emanating rootlets (black). The rootlets are organized into the basal body rows (kineties) at 5 o’clock orientation (transverse microtubules (TMs)) and 7 o’clock orientation (postciliary microtubules (PCMs)) relative to the basal body. In the disrupted region of the *MKS3*-depleted cell **(B)**, the microtubule rootlets are present and maintain the angle between them **(C)**, but do not maintain the regular orientation along the anteroposterior cell axis as shown in the control cell. Scale bars: 10 μm. The angles between the TM and the PCM were measured **(C)**, and these angles were maintained in the *MKS3*-depleted cells (35 cells from both test and control cells). Error bars are ± SD.

Figure 5 shows representatives of both *IFT88*-depleted cells (Figure 5A) and *MKS3*-depleted cells (Figure 5B). Basal bodies of the control *IFT88*-depleted cells showed organized rows and microtubule rootlets that maintained their polarity and orientation. In contrast, the representative *MKS3*-depleted cell showed twisting of a basal body row and with it a new alignment of the TM and PCM rootlets. The organized pattern of the *IFT88*-depleted cell is enlarged in Figure 5A (yellow box) and traced to show the basal bodies (red) and their microtubule rootlets (black). The same pattern is shown for the *MKS3*-depleted cell in Figure 5B (yellow box), where the orientation of the microtubule rootlets, as well as the basal bodies, can clearly be seen. The angle between the TM and PCM ribbons that emanate from the basal body was maintained in the *MKS3*-depleted cells (Figure 5C), but the orientation of the rootlets relative to the anteroposterior axis was changed. Both of the images of representative cells shown in Figure 5 are of the dorsal surfaces, and the enlarged areas are from near the dorsal midline. Note that the microtubule rootlet misalignments coincide with basal body misalignments, but not *vice versa*. The third rootlet, the striated rootlet (SR), also called the *kinetodesmal fiber*, was visualized using anti-KDF [37] and the basal bodies with anti-Glu-α-tubulin.

**Figure 6:**
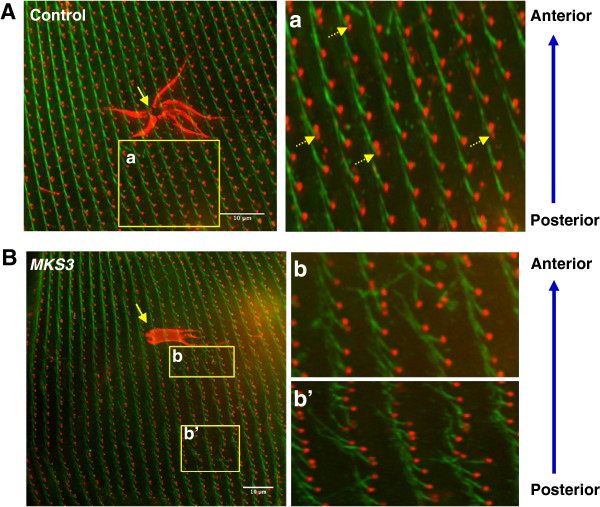
**Chaotic orientation of the striated rootlet in *MKS3***-**depleted cells.** Control cells **(A)** and *MKS3*-depleted cells **(B)** were stained with anti-Glu-α-tubulin (red; basal bodies) and anti-kinetodesmal fibers (green; striated rootlets). Yellow arrows in **(A)** and **(B)** indicate the contractile vacuoles on the dorsal surface of these cells. Striated rootlets (SRs) project from the basal bodies. In two basal body units, SRs project only from the posterior basal body (a; dotted yellow arrows). SRs project toward the anterior of the cell in a highly organized manner along the kinety. Cells depleted of *MKS3* depict chaotic organization of the SRs, which project in every direction (b and b′). Scale bar: 10 μm.

Figure 6A shows a control RNAi-fed cell with basal bodies (red) forming clear, organized rows, or kineties, along the cell surface. Emanating from the left side of each basal body is a striated rootlet (green). These fibers extend toward the anterior pole of the cell and span two or more cortical units [19]. The control cell clearly demonstrates the anterior orientation of the SRs. In the case of two basal body units, this fiber projects only from the posterior of the basal body pair (Figure 6A, yellow arrows). The large red structures in Figures 6A and 6B are the contractile vacuoles and are not the subject of this study. In the *MKS3*-depleted cell within the areas of basal body misalignment, the SRs do not always project anteriorly and often veer in oblique directions (Figure 6B(b) and 6B(b′)). The basal bodies are no longer maintained in their kinety rows, and, much like the twisted orientation of the PCMs and TMs shown in Figure 5, the SRs are chaotic in their orientations. These data, in conjunction with the TM and PCM data (Figure 5), suggest that these rootlets normally develop from the basal body, but the basal body has lost its orientation and does not maintain its position along the anteroposterior axis of the cell (see also Additional file 7: Figure S3).

### Mass spectrometry and potential interacting partners

Whole-cell extract was collected from wild-type cells, solubilized and probed using either expressed GST or expressed GST fused with the coiled-coil domain of MKS3. Samples were separated on SDS-PAGE gels and silver-stained, and the entire test (GST-MKS3 coiled-coil) and control (GST) lanes were analyzed by LC-MS/MS. We considered only those proteins unique to the test lane. In total, five proteins unique to the test sample were identified (Table 1). These proteins had a minimum of two unique peptides and included two *Paramecium* centrin-binding proteins (PtCenBP1), a sarcoendoplasmic reticulum calcium transport ATPase pump (PtSERCA1), a Ran-GTPase-activating protein and a kinetodesmal fiber protein (KdB2).

**Table 1:** **Unique proteins to the test lane from glutathione *S*-transferase-MKS3 coiled-coil domain pull-down**

**ParameciumDB accession number**	**Molecular weight**	**Peptides (control)**	**Peptides (test)**	**Name**
GSPATG00034434001	146 kDa	0	2	PtCenBP1
GSPATG00020240001	115 kDa	0	2	PtSERCA1
GSPATG00034433001	85 kDa	0	2	PtCenBP1
GSPATG00009639001	39 kDa	0	2	Ran-GTPase-activating protein 1
GSPATG00008129001	36 kDa	0	3	KdB2

## Discussion

### Reduced *MKS3* leads to abnormal and missing cilia

We expressed FLAG-tagged MKS3 protein to localize it within the *Paramecium* cell and used feeding RNAi to explore for new functions of this protein. *IFT88* served as a control for our approach because reduction of *IFT88* mRNA would inhibit ciliary transport and help to determine whether short and missing cilia are sufficient to explain the RNAi phenotype for MKS3. Both *IFT88*- and *MKS3*-depleted cells showed shortening and loss of cilia over the entire cell, except in the oral groove. These results for *MKS3* depletion are in agreement with those of Dawe *et al*. [27], who reported that small interfering RNA (siRNA) duplexes against *MKS3* caused short or missing cilia in inner medullary collecting duct cells (IMCD3). In the same study, siRNA duplexes were used against *IFT88* in IMCD3 cells, leaving more than 90% of the cells without a primary cilium. The authors concluded that loss of MKS3 disrupts polarity of centrioles and their migration to the cell surface for ciliary formation [27]. Other studies have found a variety of changes in cilia numbers and length, possibly because of differences between cell types and methods of interfering with MKS3 expression through reduction in amount or mutation [27,38-41].

We found that aspects other than short and missing cilia differed between the *Paramecium MKS3* RNAi phenotypes and *IFT88* RNAi phenotypes. For example, the cilia that remained on the *IFT88*-depleted cells appeared short but not misshapen, whereas those on *MKS3*-depleted cells were short with bulging membranes, giving them a blebby appearance, especially at the tips.

In other systems, MKS3 (TMEM67) functions as part of the filter or as a gatekeeper in the transition zone, which is the region between the basal body and the ciliary necklace [13,42-44]. Failure of transition zone function to control ciliary structure and membrane composition can lead to short and bulbous cilia [45], which is similar to our observed blebby cilia on cells depleted of *MKS3* by RNAi. Our immunofluorescence data of paramecia expressing FLAG*-MKS3* suggest that FLAG-MKS3p is in the transition zone, which in *Paramecium* has been defined as an area that spans from the epiplasm to the base of the cilium below the ciliary necklace [25,26] (Figure 1 and Additional file 5: Movie S1). The antibody we used to label basal bodies recognizes *Tetrahymena* centrin 1, which is most homologous to *Paramecium* centrin 2 and stains the full length of the *Paramecium* basal body below the cell surface [24]. The transverse section through the surface of the cell shows anti-FLAG labeling for FLAG-*MKS3* near the cell surface and at or above the distal end of the basal body. This location of MKS3p in *P. tetraurelia* is also consistent with the observations reported by Dawe *et al*. [27], who were the first to show the localization of MKS3p at the base of the primary cilium at the transition zone in IMCD3 and HEK293 cells transfected with N-terminal tagged proteins. Other groups have reported similar findings [13,46].

We propose that the depletion of MKS3p from the transition zone accounts for the loss of cilia and blebbing of the membrane of the short remaining cilia by causing a failure of the transition zone to regulate ciliary structure and membrane composition. Our data also suggest the presence of MKS3p in the distal portion of the cilium (observed by Western blotting) (Additional file 2: Figure S1). The cilia for the Western blot preparations are severed from the cell body above the ciliary necklace, which means that if MKS3p is in the cilia, the proteins on the blot come from above the transition zone [47].

### New phenotypes of *MKS3* mRNA depletion suggest interaction with basal body striated rootlets

The repetitive stereotypical rows of cortical units of *P. tetraurelia* allowed us to identify subtle deviations due to reduction of MKS3p. RNAi for *MKS3* led to basal bodies out of kinety rows on the dorsal surface, mostly at the midline. The disorganized basal bodies were in patches, mostly in clusters or, less often, in small extra rows and with misshapen morphology of cortical ridges. These phenotypes were not seen in the *IFT88*-depleted cells, indicating that the shortening and loss of cilia are not sufficient to explain these changes in *MKS3*-depleted paramecia.

Cortical units across the surface of *Paramecium* contain either one or two basal bodies (mono- or dikinetids, respectively). In preparation for cell division, basal bodies duplicate and the cell must enlarge and elongate. This first stage of division involves the conversion of all dikinetids to monokinetids [21], with the exception of those in the invariant zones at the extreme anterior and posterior ends of the cells [19,21]. This conversion of di- to monokinetids is the earliest stage in preparation for cell division, and, once complete, the cell will begin basal body duplication and formation of the fission furrow at the midline of the cell [21]. For both the conversion of di- to monokinetids and basal body duplication, where a new basal body is produced anterior to the parental basal body, the anterior basal bodies or the new basal bodies move away from the parental basal bodies using the SR as a guide, thus maintaining orderly rows [19,21]. Although basal bodies in all areas outside the invariant zones must duplicate for cell division, we did not observe a distortion of the kinety rows of basal bodies except on the dorsal side and primarily at the midline. Therefore, we propose that the *MKS3* RNAi phenotype of disorientation of basal bodies and rootlet orientations that we observed is primarily due to failure of the anterior basal body of two basal body units to migrate appropriately along the SR and maintain a straight kinety row. The conversion from di- to monokinetids occurs *prior* to basal body duplication first on the dorsal surface of the cell, where a large number of randomly distributed dikinetids exist [20], and at the midline because the anterior movement occurs in advance of basal body duplication.

**Figure 7:**
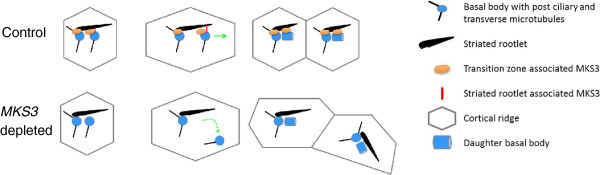
**Normal transition of a dikinetid to a monokinetid in a control cell prior to basal body duplication.** In this case, the anterior basal body of the pair is linked to and uses the striated rootlet (SR) as a guide during movement. MKS3p is shown at each basal body near the cell surface (transition zone–associated MKS3p) and also at the attachment of the anterior basal body to the SR (SR-associated MKS3p). We propose that this second location of MKS3p is temporary because MKS3p is not required as a guide once the anterior basal body has moved away from the posterior one sufficiently to develop its rootlets and foster the growth of another basal body. The lower portion of the panel diagrams the transition from a dikinetid to a monokinetid in *MKS3*-depleted cells. We propose that, in the absence of MKS3p, the anterior basal body loses its mooring and can no longer utilize the SR as a guide for migration. Without guidance, the basal body wanders out of alignment, resulting in skewed SRs, disorganized basal body rows and muddled cortical ridge organization.

The posterior basal body in the dikinetid has a cilium and full complement of rootlets (TM, PCM and SR) projecting in stereotypical orientations. The anterior basal body of the pair has no cilium and only the transverse microtubules associated with it. In addition to the rootlets, a set of three cytoskeletal nodes link the basal bodies of a dikinetid to each other and to the SR [20,21]. While moving, the anterior basal body remains linked to the SR, which extends for two or more cortical units toward the anterior and appears to act as a guide that keeps the migrating basal bodies aligned with the cortical row. Once the anterior basal body has separated from the posterior basal body, it develops a PCM and SR in addition to its preexisting TM [21]. A schematic of this process is shown in Figure 7.

We propose that misguidance in the early movement of the anterior basal body of dikinetids can account for the observed RNAi phenotype of misalignments, primarily near the dorsal midline. We also propose that all newly forming basal bodies that also use the SR as a guide could require MKS3p for their attachment to the SR. We expect that only the errors in anterior basal body movements in the dikinetics are noticed in *MKS3* RNAi-depleted cells because they occur early, before basal body duplication, and because cells stop growing and do not proceed further with basal body duplication and cell division. Indeed, these RNAi-treated cells stop growing after 24 hours of RNAi feeding (data not shown). This hypothesis of interaction of MKS3p with the SR is strongly supported by the results of a GST pull-down assay. Using the MKS3p coiled-coil domain as bait, we have identified a kinetodesmal fiber protein (KdB2:GSPATG00008129001).

MKS3p bait also pulls down two *Paramecium* centrin binding protein 1 s (PtCenBP1: GSPATG00034434001 and GSPATG00034433001), which have been shown to be the main components of the infraciliary lattice (ICL). The ICL is a contractile cortical cytoskeletal network that is nucleated from the basal body region [48,49]. An interaction of MKS3p and PtCenBP1 could help to stabilize the basal body within the cortical unit, allowing a cilium to be properly established.

GST pull-down results included Ran-GTPase-activating protein 1 (Ran-GAP:GSPATG00009639001). An interaction of RanGAP1 with MKS3p is interesting if the ciliary pore functions in a fashion similar to the nuclear pore complex, which has been suggested previously [50,51]. A Ran-GTPase/Ran-guanosine diphosphatase gradient between the ciliary and cellular compartments has been suggested to be involved in the entrance of select proteins such as Kif17 into the cilia in mammalian cells [52]. The interaction of MKS3p with RanGAP1 may prove to be a reflection of MKS3p function in the transition zone and part of the explanation for loss and deformation of cilia in MKS3 depletion.

It might be suggested that the misalignment phenotypes in *MKS3*-depleted cells is a result of inappropriate development of the SRs around the basal body. We do not favor this explanation, because in areas of disruption the PCM and TM form with a normal angle between them and KDFs develop. These results suggest that the entire basal body unit with its rootlets appears to be misdirected and not aligned with the anteroposterior axis of a kinety, as opposed to a dysfunction in SR development.

We did not identify a second location for MKS3 in our immunofluorescence studies of tagged MKS3p outside the transition zone. Although physical interactions of basal bodies and the SR were identified in structural studies [21], the transient nature of the attachments made it difficult to identify the interacting components. Our findings open up a new opportunity to dissect these transient but critical interactions.

## Conclusion

There appear to be dual roles for MKS3 in *Paramecium.* First, we have shown that MKS3p in *P. tetraurelia* is located at the cell surface near each basal body’s transition zone, where it most likely helps to filter and import (or retain) proteins into the cilia. When MKS3p from this location is reduced, cilia are lost and the cell surface and ciliary membranes become distorted. Second, a pool of MKS3 may be required in dikinetid units to guide the anterior basal body of the separating pair along the striated rootlet. A reduction in this pool of MKS3 may lead to the basal body becoming twisted and misaligned from its polarized row.

## Abbreviations

AMP: Ampicillin; DIC: Differential interference contrast; ER: Endoplasmic reticulum; EGTA: Ethyleneglycoltetraacetic acid; HEK293: Human embryonic kidney 293 cell line; HEPES: 2-[4-(2-hydroxyethyl)piperazin-1-yl]ethanesulfonic acid; IMCD3: Inner medullary collecting duct cell line; IFT: Intraflagellar transport; IPTG: Isopropyl-β-D-thiogalactopyranoside; MKS3: Meckelin; MKS: Meckel-Gruber syndrome; PCM: Postciliary microtubule; RNAi: RNA interference; SEM: Scanning electron microscopy; SD: Standard deviation; SR: Striated rootlet or kinetodesmal fiber; STDEM: Standard error of the mean; TEM: Transmission electron microscopy; TM: Transverse microtubule.

## Competing interests

The authors declare that they have no competing interests.

## Authors’ contributions

TP was responsible for the anti-2F12, anti-KDF and tubulin rootlet immunostaining data and diagrams; GST-MKS3 coiled-coil domain construct creation, expression and pull-down; LC-MS/MS analysis, figure preparation, organization, preparation and critical reading of manuscript; and project and experiment design. MSV was responsible for the creation of the *IFT88* RNAi plasmid and FLAG-*MKS3* plasmid, basal body staining, FLAG-MKS3 protein localization, SEM, TEM, database searches, manuscript preparation, figure preparation, statistical analysis and experiment and project design. JY was responsible for all plasmid injections, experiment guidance and project design and critical reading of manuscript. JVH was the Principal Investigator for the project and was responsible for experiment and project design and preparation and critical reading of the manuscript. All authors read and approved the final manuscript.

## Supplementary Material

Additional file 1: Table S1 Comparison of *Paramecium* intraflagellar transport 88 (IFT88) with other organisms. **Table S2.** Comparison of *Paramecium* meckelin (MKS3) with other organisms. Click here for file

Additional file 2: Figure S1 Alignment of the full-length *Paramecium*, mouse and human MKS3 amino acid sequences **(A)**. **(B)** Cysteine-rich domain and coiled-coil domain show conservation across all species. The *Paramecium* cysteine-rich domain shows 23% identity to both the mouse and human sequences, and the majority of the cysteines in this region are conserved across all three species. The meckelin (MKS3) coiled-coil domain of *Paramecium* shows 59% identity to the mouse MKS3 coiled-coil domain and 55% identity to the human MKS3 coiled-coil domain. For all alignments, red indicates 100% amino acid identity, green indicates an amino acid consensus match and white indicates a mismatch. Click here for file

Additional file 3: Figure S2 Alignment of the full-length *Paramecium*, mouse and human intraflagellar transport 88 (IFT88) amino acid sequences **(A)**. Four of the predicted tetratricopeptide repeat (TPR) domains of IFT88 are conserved in the *Paramecium* sequence **(B)**. TPR1 shows 44% and 42% identity, TPR2 shows 45% and 51% identity, TPR3 shows 54% and 53% identity and TPR4 shows 43% and 44% identity to the mouse and human TPR domains, respectively. For all alignments, red indicates 100% amino acid identity, green indicates an amino acid consensus match and white indicates a mismatch. Click here for file

Additional file 4: Figure S4 To help determine the localization of this protein, we examined its presence in isolated whole cilia and pure cell (pellicle) membrane from cells expressing FLAG-*MKS3* or, as a control, FLAG. The isolated proteins were then separated on SDS-PAGE gels and transferred to a nitrocellulose membrane. The nitrocellulose blots were then probed using anti-FLAG or anti-tubulin (loading control). The FLAG-MKS3 protein can be seen at 105 kDa in the cell membrane and at 105 and 77 kDa in the whole cilia (blue arrows in Figure 1C in the main text). There are nonspecific bands present in both the test and control lanes in the whole cilia blot (gray arrows in Figure 1C in the main text; approximately 107 kDa) due to the large amount of protein loaded (250 μg). Western blots developed with anti-FLAG of cell membrane and whole cilia show the FLAG-MKS3 protein in the cell membrane (blue arrowhead; approximately 105 kDa) and cilia (blue arrowheads; 105 and 77 kDa). Nonspecific bands present in both the test and control lanes are indicated by gray arrows. A representative anti-tubulin loading control blot is also shown. Click here for file

Additional file 5: Movie S1 FLAG-*MKS3*-injected cell shown in Figure 1 in the main text begins outside the cell, with its anterior side on the right. As the movie plays, each frame is one z-section, showing the FLAG stain (green) in the frame before the centrin stain (red). Notice that the FLAG stain appears before (above) the basal bodies, which are positioned slightly below the membrane, and is positioned to the side and posteriorly. Click here for file

Additional file 6 **Cells depleted of *IFT88 *and *MKS3 *were compared to cells fed the empty RNAi vector (L4440) and immunostained with anti-tubulin (Sigma-Aldrich, St Louis, MO, USA) at a 1:200 dilution as described in Materials and methods.** Cilia were measured using the DeltaVision microscopy system and softWoRx Pro software and compared using Student’s *t*-test. We measured the remaining cilia on the surfaces of three cells of each type (control and *IFT88*- and *MKS3*-depleted). Those cilia remaining on the *MKS3*- and *IFT88*-depleted cells were significantly shorter than the control cilia (*P* < 0.0001 by Student’s *t*-test). The *MKS3*- and *IFT88*-depleted cells had average cilia lengths of 3.7 ± 0.1 μm (*n* = 412 cilia) and 3.7 ± 0.2 μm (*n* = 279 cilia), respectively, compared to the control cells, whose cilia were 9.7 ± 0.1 μm (*n* = 191 cilia). Click here for file

Additional file 7: Figure S3 Images of control and *MKS3* RNAi cells stained with anti-kinetodesmal fiber (anti-KDF) (green) and anti-Glu-α-tubulin (red) show a larger section of the dorsal surface. Normal kinety and KDF alignment can be seen across the entire surface of the control cell. The *MKS3*-depleted cell shows clustering disruptions in multiple regions of the dorsal surface (yellow arrows). Click here for file
