# Supplementary material for: Reduction of meckelin leads to general loss of cilia, ciliary microtubule misalignment and distorted cell surface organization
Source: Cilia. 2014 Jan 31;3:2. doi: 10.1186/2046-2530-3-2 (PMC4124839; doi:10.1186/2046-2530-3-2)
Supplement: Additional file 1: Table S1 — Comparison of Paramecium intraflagellar transport 88 (IFT88) with other organisms. Table S2. Comparison of Paramecium meckelin (MKS3) with other organisms. [file 2046-2530-3-2-S1.docx]

**Supplemental Table 1. Comparison of *Paramecium* IFT88 with other organisms**

| **Gene (GSPATG…)** | **Nucleic acids** | **Amino acids** | **Best non-ciliate match** | **e-value** | ***Chlamydomonas***  **match (XP_001700100.1)** | **Human match**  **IFT88 Isoform 2**  **(NP_006522.2)** |
| --- | --- | --- | --- | --- | --- | --- |
| 00038505001 | 2341 | 743 | *Salmo Salar* (ACN11151) | 1.00e^-150^ | 2e^-170^  (42% identical) | 6e^-168^  (38% identical) |
| 00021390001 | 2319 | 726 | *Salmo Salar* (ACN11151) | 4.00e^-145^ | 1e^-163^  (42% identical) | 2e^-159^  (40% identical) |
| 00011771001 | 2346 | 736 | *Salmo Salar* (ACN11151) | 2.00e^-136^ | 1e^-153^  (39% identical) | 3e^-156^  (37% identical) |
| 00022644001 | 2342 | 730 | *Danio rerio* (CAQ14425) | 7.00e^-128^ | 2e^-138^  (37% Identical) | 9e^-140^  (35% Identical) |
| 00039556001 | 1727 | 566 | *Salmo Salar* (ACN11151) | 1.00e^-107^ | 9e^-116^  (39% identical) | 2e^-116^  (38% identical) |

**Supplemental Table 2. Comparison of *Paramecium* MKS3 with other organisms**

| **Gene (GSPATG…)** | **Nucleic acids** | **Amino acids** | **Best non-ciliate match** | **e-value** | ***Mus muscullus***  **(NP_808529.2)** | **Human match**  **(NP_001135773.1)** |
| --- | --- | --- | --- | --- | --- | --- |
| 00015939001 | 2906 | 951 | *Rattus norvegicus* (NP_001101386.3) | 8e^-164^  (24% identical) | 5e^-60^  (24% identical) | 2e^-56^  (25% identical) |
