# Supplementary material for: Reduction of meckelin leads to general loss of cilia, ciliary microtubule misalignment and distorted cell surface organization
Source: Cilia. 2014 Jan 31;3:2. doi: 10.1186/2046-2530-3-2 (PMC4124839; doi:10.1186/2046-2530-3-2)
Supplement: Additional file 2: Figure S1 — Alignment of the full-length Paramecium, mouse and human MKS3 amino acid sequences (A). (B) Cysteine-rich domain and coiled-coil domain show conservation across all species. The Paramecium cysteine-rich domain shows 23% identity to both the mouse and human sequences, and the majority of the cysteines in this region are conserved across all three species. The meckelin (MKS3) coiled-coil domain of Paramecium shows 59% identity to the mouse MKS3 coiled-coil domain and 55% identity to the human MKS3 coiled-coil domain. For all alignments, red indicates 100% amino acid identity, green indicates an amino acid consensus match and white indicates a mismatch. [file 2046-2530-3-2-S2.pdf]

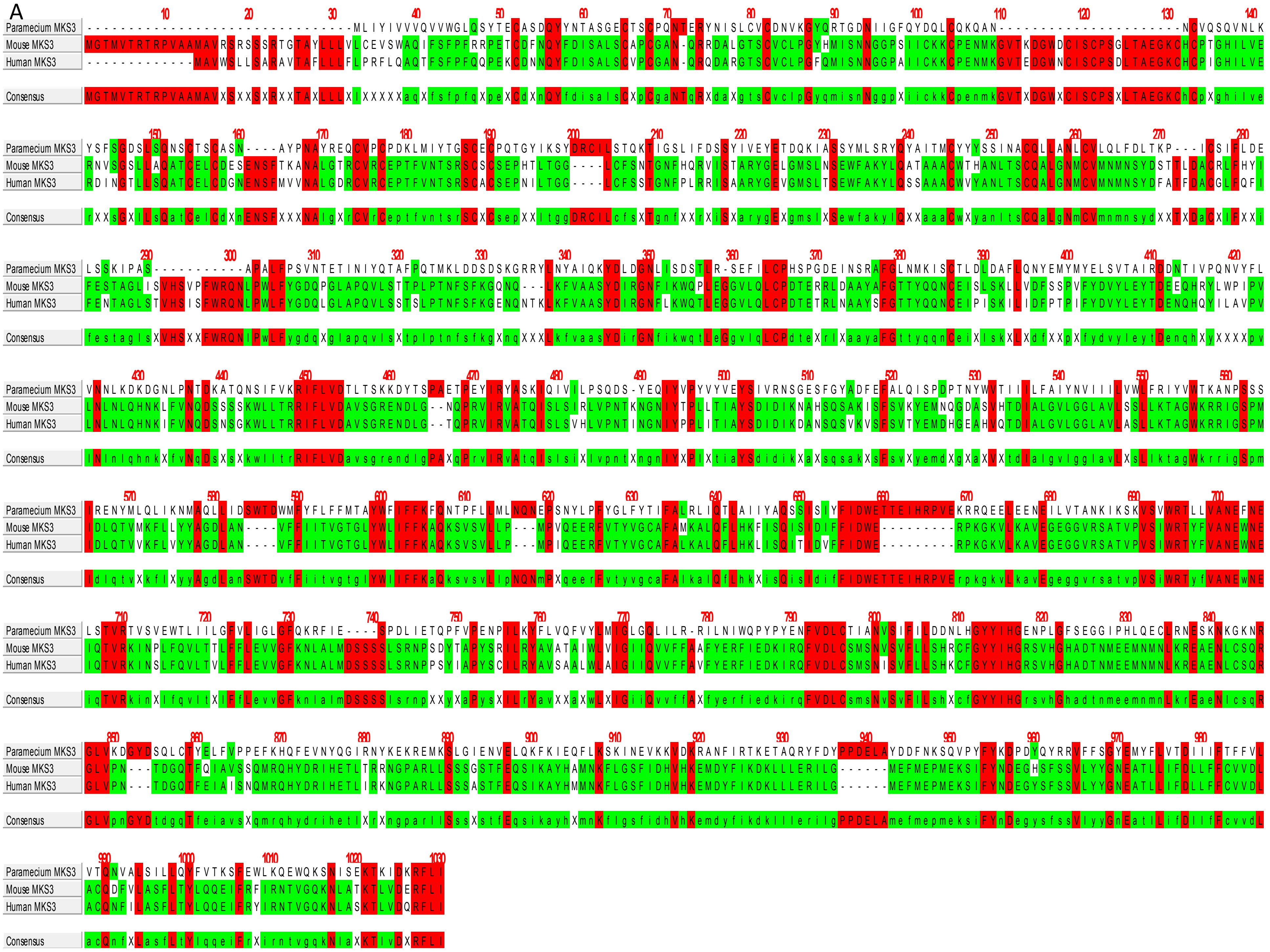
A
Paramecium MKS3
Mouse MKS3
Human MKS3
Consensus

B

Cysteine Rich Domain

	10 20 30 40 50 60 70 80
Paramecium MKS3 Cysteine Rich domain	T E C A S D Q Y Y N T A S G E C T S C P Q N T E R Y N I S L C V C D N V K G Y Q R T G D N I I G F Q Y D Q L C Q K Q A N -
Mouse MKS3 Cysteine rich domain	E T C D F N Q Y F D I S A L S C A P C G A N - Q R R D A L G T S C V C L P G Y H M I S N N G G P S I I C K K C P E N M K G V T K D G W D C I S C P S G L T A E G
Human MKS3 Cysteine Rich domain	E K C D N N Q Y F D I S A L S C V P C G A N - Q R Q D A R G T S C V C L P G F Q M I S N N G G P A I I C K K C P E N M K G V T E D G W N C I S C P S D L T A E G
Consensus	e X C d X n Q Y f d i s a l s C X p C g a N T q R X d a X g t s C v c l p G y q m i s n N g g p X i i c k k C p e n m k G V T X D G W X C I S C P S X L T A E G

	90 100 110 120 130 140
Paramecium MKS3 Cysteine Rich domain	- - N C V Q S Q V N L K Y S F S G D S L S Q N S C T S C A S N - - - - A Y P N A Y R E Q C V P C P D K L M I Y T G S C E C
Mouse MKS3 Cysteine rich domain	K C H C P T G H I L V E R N V S G S L L A Q A T C E L C D E S E N S F T K A N A L G T R C V R C E P T F V N T S R S C S C
Human MKS3 Cysteine Rich domain	K C H C P I G H I L V E R D I N G T L L S Q A T C E L C D G N E N S F M V V N A L G D R C V R C E P T F V N T S R S C A C
Consensus	K C h C p X g h i l v e r X X s G X l L s Q a t C e l C d X n E N S F X X X N A l g X r C V r C e p t f v n t s r S C X C

Coiled Coil Domain

	10 20 30
Paramecium MKS3 Coiled Coil domain	N F V D L C T I A N V S I F I L D D N L H G Y Y I H G E N P
Mouse MKS3 Coiled Coil domain	Q F V D L C S M S N V S V F L L S H R C F G Y Y I H G R S V
Human MKS3 Coiled Coil domain	Q F V D L C S M S N I S V F L L S H K C F G Y Y I H G R S V
Consensus	q F V D L C s m s N v S v F l L s h c f G Y Y I H G r s v
