# Supplementary material for: Reduction of meckelin leads to general loss of cilia, ciliary microtubule misalignment and distorted cell surface organization
Source: Cilia. 2014 Jan 31;3:2. doi: 10.1186/2046-2530-3-2 (PMC4124839; doi:10.1186/2046-2530-3-2)
Supplement: Additional file 3: Figure S2 — Alignment of the full-length Paramecium, mouse and human intraflagellar transport 88 (IFT88) amino acid sequences (A). Four of the predicted tetratricopeptide repeat (TPR) domains of IFT88 are conserved in the Paramecium sequence (B). TPR1 shows 44% and 42% identity, TPR2 shows 45% and 51% identity, TPR3 shows 54% and 53% identity and TPR4 shows 43% and 44% identity to the mouse and human TPR domains, respectively. For all alignments, red indicates 100% amino acid identity, green indicates an amino acid consensus match and white indicates a mismatch. [file 2046-2530-3-2-S3.pdf]

A

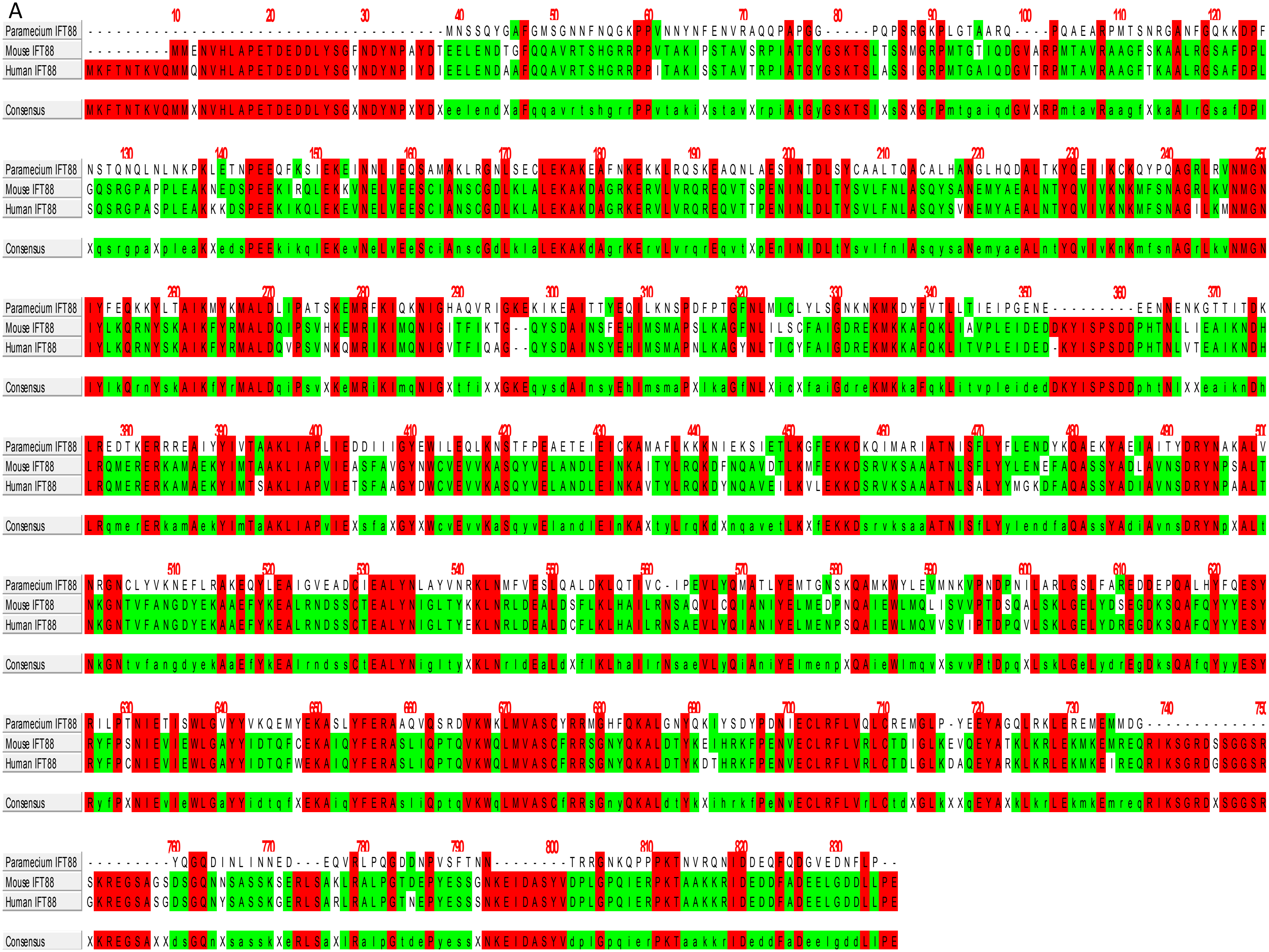

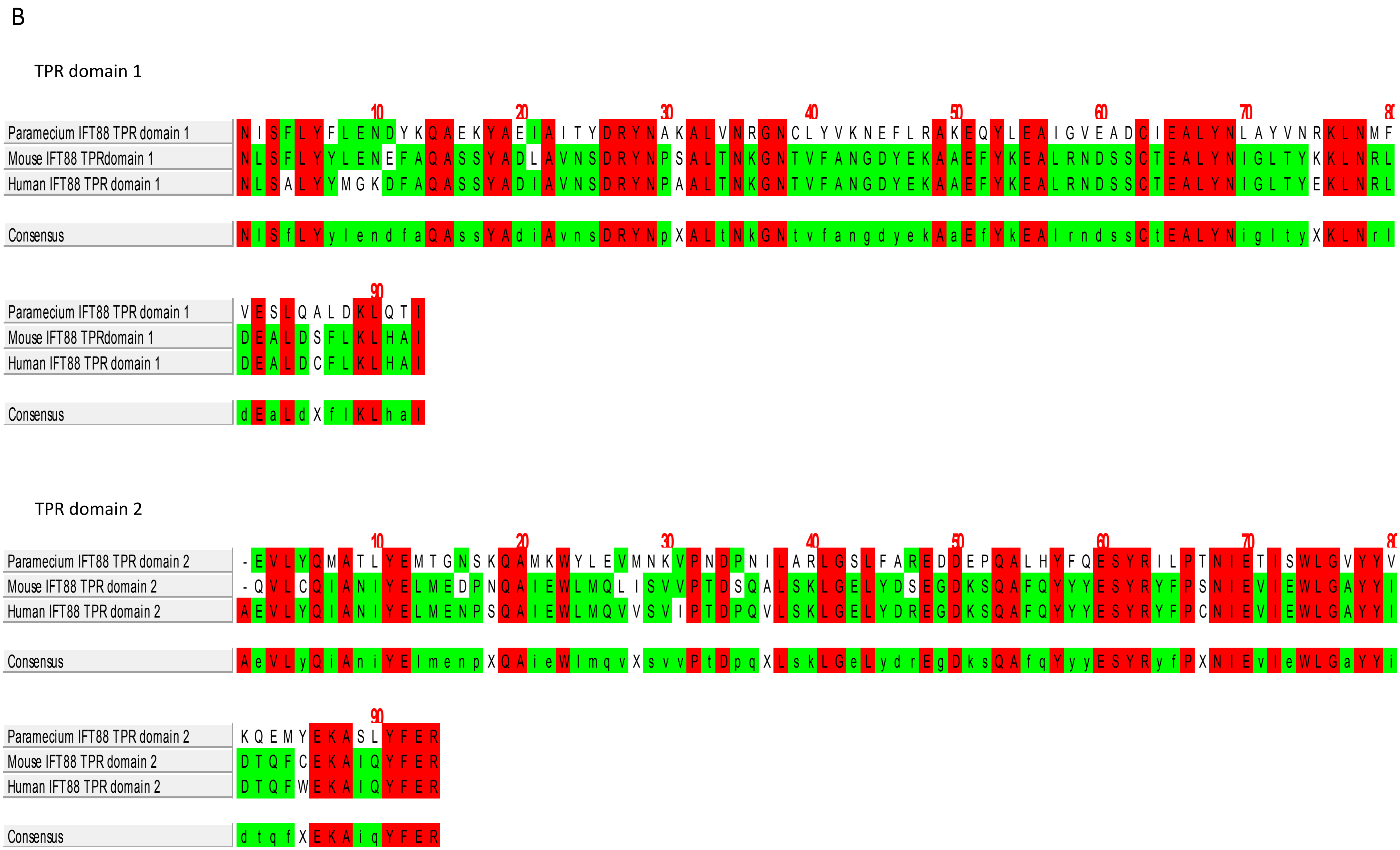
B
TPR domain 1
Paramecium IFT88 TPR domain 1 N I S F L Y F L E N D Y K Q A E K Y A E I A I T Y D R Y N A K A L V N R G N C L Y V K N E F L R A K E Q Y L E A I G V E A D C I E A L Y N L A Y V N R K L N M F
Mouse IFT88 TPRdomain 1 N L S F L Y Y L E N E F A Q A S S Y A D L A V N S D R Y N P S A L T N K G N T V F A N G D Y E K A A E F Y K E A L R N D S S C T E A L Y N I G L T Y K K L N R L
Human IFT88 TPR domain 1 N L S A L Y Y M G K D F A Q A S S Y A D I A V N S D R Y N P A A L T N K G N T V F A N G D Y E K A A E F Y K E A L R N D S S C T E A L Y N I G L T Y E K L N R L
Consensus N I S f L Y y l e n d f a Q A s s Y A d i A v n s D R Y N p X A L t N k G N t v f a n g d y e k A a E f Y k E A l r n d s s C t E A L Y N i g l t y X K L N r l
Paramecium IFT88 TPR domain 1 V E S L Q A L D K L Q T I
Mouse IFT88 TPRdomain 1 D E A L D S F L K L H A I
Human IFT88 TPR domain 1 D E A L D C F L K L H A I
Consensus d E a L d X f l K L h a I
TPR domain 2
Paramecium IFT88 TPR domain 2 - E V L Y Q M A T L Y E M T G N S K Q A M K W Y L E V M N K V P N D P N I L A R L G S L F A R E D D E P Q A L H Y F Q E S Y R I L P T N I E T I S W L G V Y Y V
Mouse IFT88 TPR domain 2 - Q V L C Q I A N I Y E L M E D P N Q A I E W L M Q L I S V V P T D S Q A L S K L G E L Y D S E G D K S Q A F Q Y Y Y E S Y R Y F P S N I E V I E W L G A Y Y I
Human IFT88 TPR domain 2 A E V L Y Q I A N I Y E L M E N P S Q A I E W L M Q V V S V I P T D P Q V L S K L G E L Y D R E G D K S Q A F Q Y Y Y E S Y R Y F P C N I E V I E W L G A Y Y I
Consensus A e V L y Q i A n i Y E l m e n p X Q A i e W l m q v X s v v P t D p q X L s k L G e L y d r E g D k s Q A f q Y y y E S Y R y f P X N I E v I e W L G a Y Y i
Paramecium IFT88 TPR domain 2 K Q E M Y E K A S L Y F E R
Mouse IFT88 TPR domain 2 D T Q F C E K A I Q Y F E R
Human IFT88 TPR domain 2 D T Q F W E K A I Q Y F E R
Consensus d t q f X E K A i q Y F E R

TPR domain 3

	10 20 30 40 50 60 70 80
Paramecium IFT88 TPR domain 3	- L G V Y Y V K Q E M Y E K A S L Y F E R A A Q V Q S R D V K W K L M V A S C Y R R M G H F Q K A L G N Y Q K I Y S D Y P D N I E C L R F L V Q L C R E M G L P
Mouse IFT88 TPR domain 3	- L G A Y Y I D T Q F C E K A I Q Y F E R A S L I Q P T Q V K W Q L M V A S C F R R S G N Y Q K A L D T Y K E I H R K F P E N V E C L R F L V R L C T D I G L K
Human IFT88 TPR domain 3	W L G A Y Y I D T Q F W E K A I Q Y F E R A S L I Q P T Q V K W Q L M V A S C F R R S G N Y Q K A L D T Y K D T H R K F P E N V E C L R F L V R L C T D L G L K
Consensus	W L G a Y Y i d t q f X E K A i q Y F E R A s l i Q p t q V K W q L M V A S C f R R s G n y Q K A L d t Y k X i h r k f P e N v E C L R F L V r L C t d X G L k

	90
Paramecium IFT88 TPR domain 3	- Y E E Y A G Q L R -
Mouse IFT88 TPR domain 3	E V Q E Y A T K L K R
Human IFT88 TPR domain 3	D A Q E Y A R K L K -
Consensus	X X q E Y A X k L k R

TPR domain 4

	10 20 30 40 50 60 70 80
Paramecium IFT88 TPR domain 4	N M G N I Y F E Q K K Y L T A I K M Y K M A L D L I P A T S K E M R F K I Q K N I G H A Q V R I G K E K I K E A I T T Y E Q I L K N S P D F P T G F N L M I C L
Mouse IFT88 TPR domain 4	N M G N I Y L K Q R N Y S K A I K F Y R M A L D Q I P S V H K E M R I K I M Q N I G I T F I K T G - - Q Y S D A I N S F E H I M S M A P S L K A G F N L I L S C
Human IFT88 TPR domain 4	N M G N I Y L K Q R N Y S K A I K F Y R M A L D Q V P S V N K Q M R I K I M Q N I G V T F I Q A G - - Q Y S D A I N S Y E H I M S M A P N L K A G Y N L T I C Y
Consensus	N M G N I Y l k Q r n Y s k A I K f Y r M A L D q i P s v X K e M R i K I m q N I G X t f i X X G K E q y s d A I n s y E h I m s m a P X l k a G f N L X i c X

	90 100
Paramecium IFT88 TPR domain 4	Y L S G N K N K M K D Y F V T L L T I E
Mouse IFT88 TPR domain 4	F A I G D R E K M K K A F Q K L I A V P
Human IFT88 TPR domain 4	F A I G D R E K M K K A F Q K L I T V P
Consensus	f a i G d r e K M K k a F q k L i t v p
