# Supplementary figures and images for: Reduction of meckelin leads to general loss of cilia, ciliary microtubule misalignment and distorted cell surface organization
Source: Cilia. 2014 Jan 31;3:2. doi: 10.1186/2046-2530-3-2 (PMC4124839; doi:10.1186/2046-2530-3-2)

**Cell Membrane**

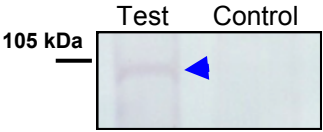

**Tubulin Loading Control**

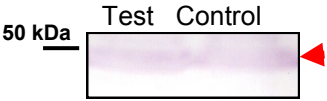

**Cilia**

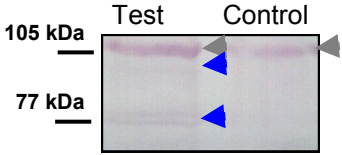

Supplement: Additional file 4: Figure S4 — To help determine the localization of this protein, we examined its presence in isolated whole cilia and pure cell (pellicle) membrane from cells expressing FLAG-MKS3 or, as a control, FLAG. The isolated proteins were then separated on SDS-PAGE gels and transferred to a nitrocellulose membrane. The nitrocellulose blots were then probed using anti-FLAG or anti-tubulin (loading control). The FLAG-MKS3 protein can be seen at 105 kDa in the cell membrane and at 105 and 77 kDa in the whole cilia (blue arrows in Figure 1C in the main text). There are nonspecific bands present in both the test and control lanes in the whole cilia blot (gray arrows in Figure 1C in the main text; approximately 107 kDa) due to the large amount of protein loaded (250 μg). Western blots developed with anti-FLAG of cell membrane and whole cilia show the FLAG-MKS3 protein in the cell membrane (blue arrowhead; approximately 105 kDa) and cilia (blue arrowheads; 105 and 77 kDa). Nonspecific bands present in both the test and control lanes are indicated by gray arrows. A representative anti-tubulin loading control blot is also shown. [file 2046-2530-3-2-S4.pdf]

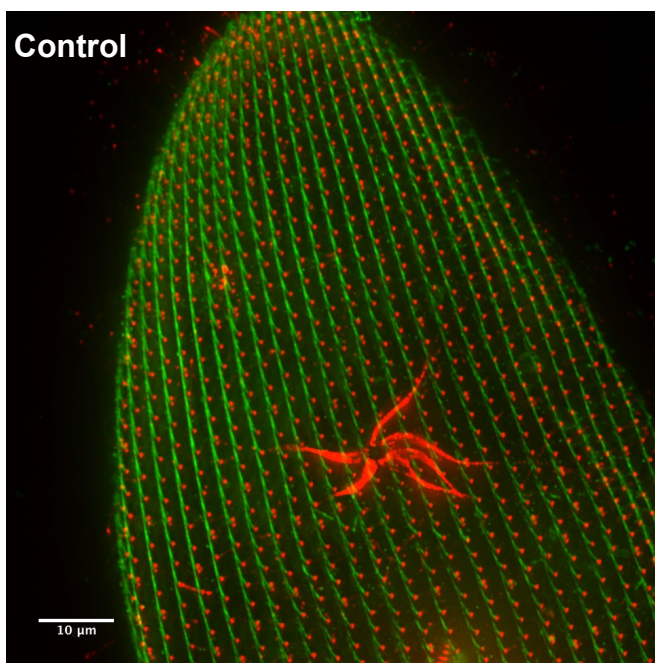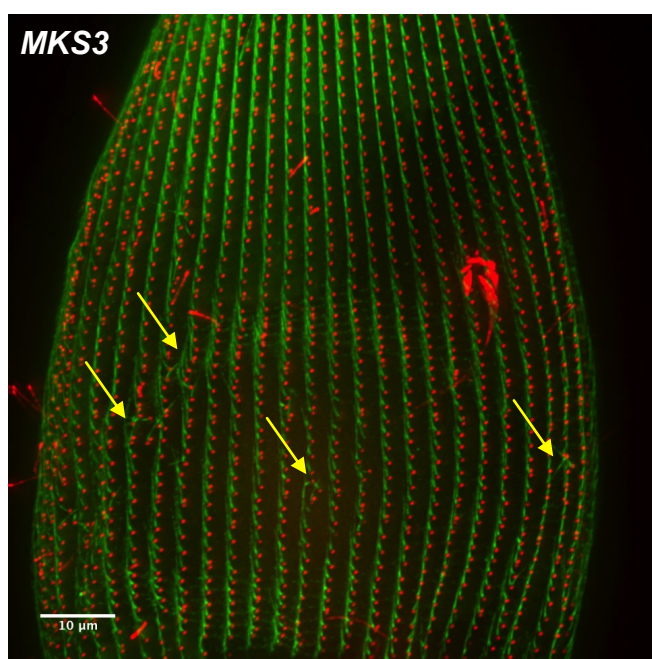

Supplement: Additional file 7: Figure S3 — Images of control and MKS3 RNAi cells stained with anti-kinetodesmal fiber (anti-KDF) (green) and anti-Glu-α-tubulin (red) show a larger section of the dorsal surface. Normal kinety and KDF alignment can be seen across the entire surface of the control cell. The MKS3-depleted cell shows clustering disruptions in multiple regions of the dorsal surface (yellow arrows). [file 2046-2530-3-2-S7.pdf]
