# Supplementary material for: Reduction of meckelin leads to general loss of cilia, ciliary microtubule misalignment and distorted cell surface organization
Source: Cilia. 2014 Jan 31;3:2. doi: 10.1186/2046-2530-3-2 (PMC4124839; doi:10.1186/2046-2530-3-2)
Supplement: Additional file 6 — Cells depleted of IFT88 and MKS3 were compared to cells fed the empty RNAi vector (L4440) and immunostained with anti-tubulin (Sigma-Aldrich, St Louis, MO, USA) at a 1:200 dilution as described in Materials and methods. Cilia were measured using the DeltaVision microscopy system and softWoRx Pro software and compared using Student’s t-test. We measured the remaining cilia on the surfaces of three cells of each type (control and IFT88- and MKS3-depleted). Those cilia remaining on the MKS3- and IFT88-depleted cells were significantly shorter than the control cilia (P < 0.0001 by Student’s t-test). The MKS3- and IFT88-depleted cells had average cilia lengths of 3.7 ± 0.1 μm (n = 412 cilia) and 3.7 ± 0.2 μm (n = 279 cilia), respectively, compared to the control cells, whose cilia were 9.7 ± 0.1 μm (n = 191 cilia). [file 2046-2530-3-2-S6.docx]

**Additional File 6.**

Cells depleted of *IFT88* and *MKS3* were compared to cells fed the empty RNAi vector (L4440) and immunostained with Anti-Tubulin (Sigma, St. Louis, MO, USA) at a 1:200 dilution as described in the Materials and Methods. Cilia were measured using the DeltaVision Microscopy system and SoftWoRx® Pro software and compared using a Students *t*-test. We measured the remaining cilia on the surfaces of three cells of each type (Control and *IFT88* and *MKS3* depleted). Those cilia remaining on the *MKS3* and *IFT88* depleted cells were significantly shorter than the control cilia (*t*-test, P<0.0001). The *MKS3* and *IFT88* depleted cells had average cilia lengths of 3.7 ± 0.1 µm (412 cilia) and 3.7 ± 0.2 µm (279 cilia), respectively, compared to the control cells, whose cilia were 9.7 ± 0.1 µm (191 cilia).
